# Clinical features and outcome of *Aeromonas sobria* bacteremia in pediatric and adult patients with hematologic malignancies: A single-center retrospective study in Peru

**DOI:** 10.1371/journal.pone.0255910

**Published:** 2021-08-11

**Authors:** Bryan Valcarcel, Gabriel De-la-Cruz-Ku, Luis Malpica, Daniel Enriquez-Vera

**Affiliations:** 1 Facultad de Ciencias de la Salud, Escuela de Medicina Humana, Universidad Científica del Sur, Lima, Perú; 2 Division of Cancer Medicine, Department of Lymphoma and Myeloma, The University of Texas MD Anderson Cancer Center, Houston, TX, United States of America; 3 Departmento de Hematología y Medicina Oncológica, Instituto Nacional de Enfermedades Neoplásicas, Lima, Perú; University of Malaya Faculty of Medicine, MALAYSIA

## Abstract

**Background:**

Previous studies have found that healthcare-associated bacteremia (HAB) by *Aeromonas* species is associated with mortality. However, there is limited data on this outcome in patients with hematologic malignancies. This study aimed to identify the clinical features of patients with malignant hematologic diseases diagnosed with *Aeromonas sobria* bacteremia and to evaluate whether the type of bacteremia, community-acquired bacteremia (CAB) or HAB, is associated with mortality.

**Methods:**

We retrospectively reviewed the clinical records of pediatric and adult patients between January 2000 and December 2017. Clinical characteristics were compared between CAB and HAB. Additionally, we stratified based on age group. Survival outcomes were assessed with Kaplan-Meier curves and a multivariate Cox regression analysis.

**Results:**

A total of 37 patients (median age 24 years) were identified; 23 (62%) had HAB and 14 (38%) had CAB. Overall, the most common presenting symptom was abdominal pain (41%). Acute lymphoblastic leukemia (n = 12/15, 80%) and acute myeloid leukemia (n = 8/22, 36%) were the primary hematologic malignancies in pediatric and adult patients, respectively. CAB patients had worse overall survival (OS) rates at 30 days in all (43% versus HAB 91%, p = 0.006) and adult patients (30% versus HAB 92%, p = 0.002). Cox regression analysis found that quick Sequential Organ Failure Assessment and CAB were statistically significant factors associated with mortality. Low antimicrobial-resistant was noted, except for ciprofloxacin (n = 5/37, 14%).

**Conclusion:**

Our study found a worse OS among patients with hematologic malignancies and CAB by *Aeromonas sobria*. Our results suggest that patients with CAB present with a worse disease severity. These findings should aid clinicians to determine the survival prognosis in this population.

## Introduction

*Aeromonas* species, a gram-negative heterotrophic bacteria mainly found in warm climates, cause human disease through contamination of water, seafood, meat, and vegetables [1, 2]. Incidence of *Aeromonas* infection varies from 20 to 76 cases per 1,000,000 persons. The most common species isolated are *A*. *hydrophila*, *A*. *sobria*, and *A*. *caviae*, accounting for about 86%, 11%, and 3% of the cases, respectively [1, 3, 4].

These bacteria cause a broad spectrum of human infections, such as gastrointestinal tract infection, septicemia, acute respiratory tract infection, soft-tissue infection, among others [5–7]. Although the disease is more commonly seen in immunocompromised individuals, it can develop in immunocompetent hosts [6]. Factors associated with mortality by *A*. *sobria* include an APACHE II score of 20 or more points, thrombocytopenia, and the presence of diarrhea [8]. Similarly, other related factors include hypotension, impaired renal function, liver cirrhosis, lower limb infection, and altered consciousness [9–11].

Nosocomial infection is a typical factor for poor prognosis and mortality, especially in patients with decreased immunity [12]. Similarly, a previous study in patients with *A*. *sobria* infection found that healthcare-associated bacteremia (HAB) was related to mortality compared to community-acquired bacteremia (CAB) [13]. However, there is scarce literature on this association in patients with hematologic malignancies and *A*. *sobria* bacteremia [8, 9, 14]. This population is commonly immunocompromised, and outcomes might differ from previous studies that included patients with a variety of comorbidities apart from hematological malignancies. Therefore, the present study aims to describe the clinical features and outcome of patients with malignant hematologic diseases diagnosed with *A*. *sobria* bacteremia and to identify the factors associated with survival in this population. Specifically, we assess whether the type of bacteremia (CAB or HAB) is related to mortality in all patients and by age group (pediatric and adults) stratification.

## Materials and methods

### Patients and inclusion/exclusion criteria

We conducted a retrospective study of pediatric and adult patients diagnosed with malignant hematologic diseases and *A*. *sobria* bacteremia at the National Institute of Neoplastic Diseases (*Instituto Nacional de Enfermedades Neoplásicas*–INEN) in Lima, Peru, between January 2000 and December 2017. Malignant hematologic diseases included in this study were: the acute phase of myeloid and lymphoid leukemias, high-grade lymphomas (i.e., diffuse large B-cell lymphoma, or Burkitt lymphoma), and multiple myeloma. Patients developed bacteremia by *A*. *sobria* while on active cancer treatment or within 30 days from their last dose. Bacteremia was diagnosed by clinical and laboratory findings, with *A*. *sobria* identified in blood cultures. We excluded patients with a previous history of a bone marrow transplant, solid tumors, and those in which outcomes were not assessable (e.g., lost to follow-up or incomplete medical records). Medical records were identified using the databases from the Microbiology Department and the Hematology and Medical Oncology Department.

### Study variables

We defined pediatric patients to those aged 0–19 years old, based on the recommendation of the International Agency for Research on Cancer [15]. CAB was defined as the presence of clinical symptoms 72 hours before hospital admission, while HAB was defined as the presence of symptoms during hospitalization or the development of symptoms within 72 hours after hospital discharge. Data can be found in the following link: https://github.com/valcarcelb/AeroS. Patients were followed for 30 days after isolation of *A*. *sobria* from the bloodstream. Grade 3 to 4 cytopenias (i.e. leukopenia, anemia, and thrombocytopenia) were defined as absolute leucocyte count of ≤1.0 × 10^3^ leukocytes/μL, hemoglobin <8 g/dL, and platelets <50 × 10^3^ cells/μL, respectively. Death by *A*. *sobria* was defined as the one occurring within the first 30 days after its isolation from the bloodstream, or if death occurred during the acute course of sepsis with post-mortem isolation of *A*. *sobria*. Infection resolution was considered when there was an improvement in clinical symptoms coupled to negative blood cultures for two consecutive times. Cancer-directed therapy response of hematologic malignancies (complete response, partial response, or disease progression) was assessed using established clinical guidelines [16–18].

### Statistical analysis

We performed descriptive statistics to present the characteristics of the patients. Age had a non-normally distribution based on the visual assessment of histograms and the Shapiro-Wilk test (p = 0.003). Therefore, we present the age at diagnosis using the median and the range. Categorical variables are shown as frequency and percentages. Based on the low expected count, categorical variables were compared using Fisher’s exact test and the age with the Mann-Whitney U test.

The influence of the type of bacteremia (CAB and HAB) was assessed using a multivariate Cox regression analysis and the results are reported with Hazard Ratios (HRs). The model includes variables that were associated with mortality in previous studies [9, 11], such as age and clinical characteristics, and factors that we considered relevant in the clinical course of bacteremia in patients with hematologic malignancies. We evaluate the effect of shock at presentation in the multivariate model for overall survival; however, the proportional hazard assumption was violated (global test: p = 0.021). Because the database has few observations, we could not perform a stratified analysis based on the presence of shock at diagnosis. The quick Sequential Organ Failure Assessment (qSOFA) score was used as a surrogate to adjust for the severity of clinical presentation. Our final model includes age group (adult vs. pediatric), comorbidities (yes vs. no), qSOFA score (≥2 vs. 0–1), type of bacteremia (CAB vs. HAB). This model did not violate the hazard assumption based on the visual assessment of the Schoenfeld residuals and the global test (p = 0.350).

We estimated the survival probabilities in all patients and by age group stratification using the Kaplan-Meier method. Survival curves between groups (HAB vs. CAB) were compared with the Log-rank test. Although we did not find an interaction between age group and type of bacteremia on the mortality outcome (p = 0.428), we stratified based on age group because of the different clinical presentation and disease outcome that is typically found between pediatric and adult patients. Statistical significance was considered with a p-value of less than 0.05. We used the R software for analysis.

### Ethical statement

The Institutional Review Board (IRB) of the INEN approved the development of this study with the code ‘INEN 18–25’. Because of the retrospective design, the IRB waived the need for informed consent. We censored the patients’ personal information during the data extraction from the medical records by assigning them numbers for identification. The data extraction was between January 2018 –April 2018.

## Results

### Clinical features in the overall population

A total of 37 patients with malignant hematologic diseases and *A*. *sobria* isolation from the bloodstream were included. Table 1 summarizes the clinical features of these patients and according to the type of bacteremia. The median age at the time of bacteremia diagnosis was 24 years (range: 2–74 years), with 22 (59%) patients being adults (≥20 years old). Most patients were diagnosed with acute lymphoblastic leukemia (n = 19/37, 51%) and acute myeloblastic leukemia (n = 10/37, 27%). Abdominal pain (41%) was the most common presenting symptom, followed by diarrhea (30%) and pain in the lower limbs (22%). A total of 82% (n = 27/33) patients presented with fever (temperature of >100.4°F or 38°C).

**Table 1:** Clinical characteristics of patients with hematological malignancies and *Aeromonas sobria* bacteremia according to the type of bacteremia.

Characteristics	All patients, n (%)	Community-acquired bacteremia, n (%)	Healthcare-associated bacteremia, n (%)	P-value
Number of patients	37	14	23	
Age, median (range)	24 (2–74)	58 (2–74)	20 (3–70)	0.028
Age group				0.314
Pediatric	15 (41)	4 (29)	11 (48)	
Adult	22 (59)	10 (71)	12 (52)	
Sex				0.101
Female	17 (46)	9 (64)	8 (35)	
Male	20 (54)	5 (36)	15 (65)	
Comorbidities	10 (27)	6 (43)	4 (17)	0.132
Hematologic malignancy				0.360
Acute lymphoblastic leukemia	19 (51)	6 (43)	13 (57)	
Acute myeloid leukemia	10 (27)	3 (21)	7 (30)	
High grade lymphoma	7 (19)	4 (29)	3 (13)	
Multiple myeloma	1 (3)	1 (7)	0 (0)	
Clinical symptoms				
Abdominal pain	15 (41)	5 (36)	10 (43)	0.738
Diarrhea	11 (30)	4 (29)	7 (30)	1.000
Pain in lower limbs	8 (22)	2 (14)	6 (26)	0.683
Anemia	13 (35)	8 (57)	5 (22)	0.039
Leukopenia	29 (78)	9 (64)	20 (87)	0.108
Thrombocytopenia	29 (78)	11 (79)	18 (78)	1.000
qSOFA score				0.182
0–1	25 (68)	7 (50)	18 (78)	
2–3	9 (24)	5 (36)	4 (18)	
Missing	3 (8)	2 (14)	1 (4)	
Primary site of sepsis				1.000
Gastrointestinal tract	23 (62)	9 (64)	14 (61)	
Osteoarticular system	7 (19)	3 (22)	4 (17)	
Not determined	7 (19)	2 (14)	5 (22)	
Septic shock	11 (30)	8 (57)	3 (13)	0.008
Antibiotics				
Meropenem	24 (65)	9 (64)	15 (65)	1.000
Ceftazidime	14 (38)	3 (21)	11 (48)	0.166
Amikacin	11 (30)	3 (21)	8 (35)	0.477
Colistin	3 (8)	0 (0)	3 (13)	0.275
Piperacillin-tazobactam	2 (5)	1 (7)	1 (4)	1.000
Meropenem + vancomycin	22 (59)	9 (64)	13 (57)	0.738
Ceftazidime + amikacin	10 (27)	3 (21)	7 (30)	0.710
Cancer treatment response				0.138
Complete response	20 (54)	5 (36)	15 (65)	
Partial response	3 (8)	1 (7)	2 (9)	
Disease progression	14 (38)	8 (57)	6 (26)	
Mortality at 30 days	10 (27)	8 (57)	2 (9)	0.002

qSOFA, Quick Sequential Organ Failure Assessment.

The source of bacteremia was identified in 30 (81%) patients, of whom 77% (n = 23/30) had the gastrointestinal tract as the primary source. Eleven (30%) patients presented with septic shock at diagnosis. Grade 3 and 4 leukopenia, thrombocytopenia, and anemia were found in 78%, 78%, and 35%, respectively. All patients received empiric antimicrobial therapy at the time of diagnosis, with the antimicrobial regimen modified according to the results of the antimicrobial susceptibility testing. Meropenem was the most common empiric antibiotic used (n = 24/37, 65%). Regarding the treatment response of hematologic malignancies, most patients developed bacteremia while on complete remission (n = 20/37, 54%) as opposed to patients in partial response (n = 3/37, 8%) or in disease progression (n = 14/37, 38%).

### Clinical features according to the type of bacteremia (hospital versus community-acquired)

Bacteremia by *A*. *sobria* was most frequently found in patients with HAB (n = 23/37, 62%) compared to CAB (n = 14/37, 38%). Patients with CAB more frequently presented with septic shock (n = 8/14, 57% versus HAB n = 3/23, 13%; p = 0.008) and had a higher fatality rate (n = 8/14, 57% versus HAB n = 2/23, 9%; p = 0.002) compared to HAB patients (Table 1). There was no difference in the frequency of grade 3–4 leukopenia (CAB n = 9/14, 64% versus HAB n = 20/23, 87%; p = 0.108) and thrombocytopenia (CAB n = 11/14, 79% versus HAB n = 18/23, 78%; p = 1.000) between both groups at the time of bacteremia diagnosis.

### Clinical features according to age group

Table 2 summarizes the clinical features of patients according to age group. There were 15 (41%) pediatric patients with a median age of 11 years (range: 2–19) and 22 (59%) adult patients with a median age of 52.5 (range: 20–74). Most pediatric patients had a diagnosis of acute lymphoblastic leukemia compared to adult patients (80% versus 32%; p = 0.028), the latter with a more comparable distribution in the type of hematologic malignancies encountered. History of asthma was reported in one pediatric patient, while seven adults reported at least one medical history related to hypertension, chronic kidney disease, diabetes mellitus, or gastritis. Both age groups had low qSOFA scores at the time of bacteremia diagnosis (score 0–1: pediatric n = 9/15, 60% versus adult n = 16/22, 73%) (Table 2). However, septic shock was more frequently seen in adults as opposed to pediatric patients, without statistical significance (41% vs. 13%, respectively; p = 0.141).

**Table 2:** Clinical characteristics of patients with hematological malignancies and *Aeromonas sobria* bacteremia according to age group.

Characteristics	Pediatric patients, n (%)	Adult patients, n (%)	P-value
Number of patients	15	22	
Sex			0.315
Female	5 (33)	12 (55)	
Male	10 (67)	10 (45)	
Comorbidities	3 (20)	7 (32)	0.481
Hematologic malignancy			0.028
Acute lymphoblastic leukemia	12 (80)	7 (32)	
Acute myeloid leukemia	2 (13)	8 (36)	
High-grade lymphoma	1 (7)	6 (27)	
Multiple myeloma	0 (0)	1 (5)	
Clinical symptoms			
Abdominal pain	8 (53)	7 (32)	0.307
Diarrhea	4 (27)	7 (32)	1.000
Pain in lower limbs	5 (33)	3 (14)	0.228
Anemia	4 (27)	9 (41)	0.491
Leukopenia	13 (87)	16 (73)	0.575
Thrombocytopenia	12 (80)	17 (77)	1.000
qSOFA score			0.660
0–1	9 (60)	16 (73)	
2–3	4 (27)	5 (23)	
Missing	2 (13)	1 (4)	
Primary site of sepsis			0.393
Gastrointestinal system	11 (73)	12 (55)	
Osteoarticular system	3 (20)	4 (18)	
Not determined	1 (7)	6 (27)	
Septic shock	2 (13)	9 (41)	0.141
Antibiotics			
Meropenem	10 (67)	14 (64)	1.000
Ceftazidime	6 (40)	8 (36)	1.000
Amikacin	3 (20)	8 (36)	0.466
Colistin	1 (7)	2 (9)	1.000
Piperacillin-tazobactam	1 (7)	1 (5)	1.000
Meropenem + vancomycin	8 (53)	14 (64)	0.734
Ceftazidime + amikacin	3 (20)	7 (32)	0.481
Cancer treatment response			0.173
Complete response	10 (67)	10 (45)	
Partial response	2 (13)	1 (5)	
Disease progression	3 (20)	11 (50)	
Bacteremia type			0.314
Community-acquired	4 (27)	10 (45)	
Healthcare-associated	11 (73)	12 (55)	
Mortality at 30 days	2 (13)	8 (36)	0.153

qSOFA, Quick Sequential Organ Failure Assessment.

### Survival outcomes

**Fig 1:**
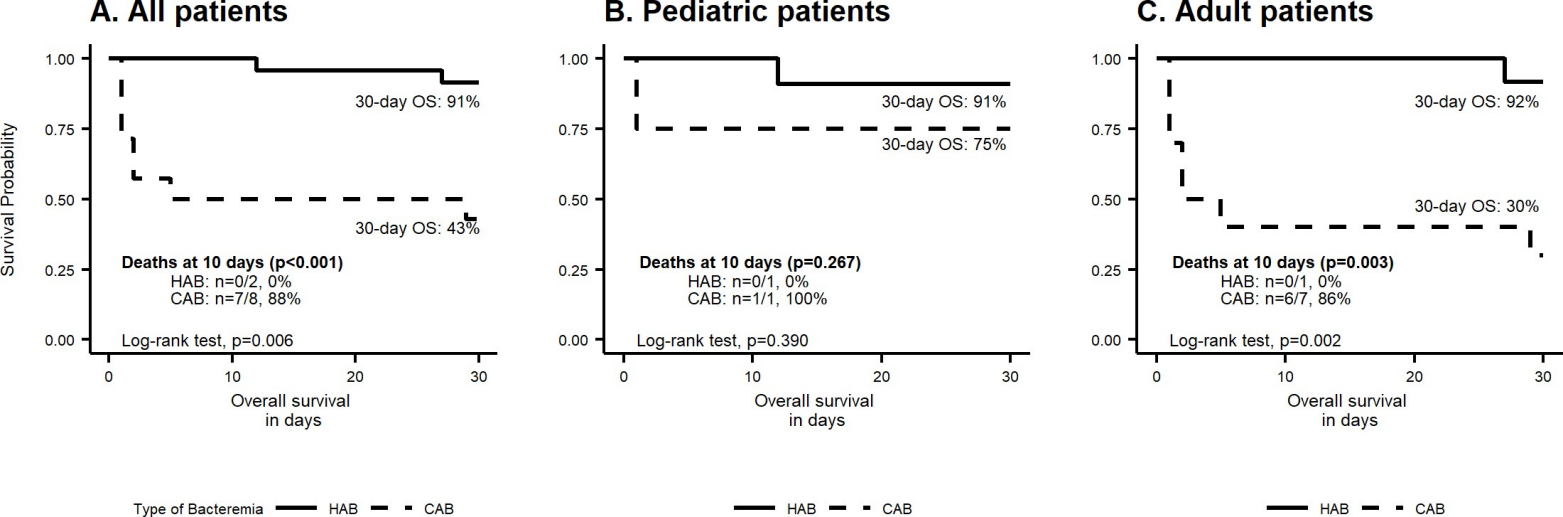
Kaplan-Meier analysis of the overall survival between the type of bacteremia in the total population (A) pediatric (B), and adult patients (C). OS, Overall survival; HAB, Healthcare-associated bacteremia; CAB, Community-acquired bacteremia.

The overall case-fatality rate was 27% (n = 10). Subjects with CAB had a higher fatality rate at 30-days compared to HAB (n = 8/14, 57% versus n = 2/23, 9%, respectively; p = 0.002) (Table 1). When stratified by age group, adults had a higher fatality rate at 30-days compared to pediatric patients, without statistical significance (36% versus 13%, respectively; p = 0.153) (Table 2). To explore the effect of *A*. *sobria* bacteremia on survival, we plotted Kaplan-Meier curves according to the type of bacteremia in the total population (Fig 1A), pediatric (Fig 1B), and adult (Fig 1C) patients. In all and adult patients, individuals with CAB had worse overall survival at 30 days (43% versus 91%, p = 0.006 and 30% versus 92%, p = 0.002, respectively). Most deaths occurred during the first ten days after isolation of *A*. *sobria* in all (n = 7/8, 88% versus n = 0/2, 0%; p<0.001) and adult patients (n = 6/7, 86% versus n = 0/1, 0%, p = 0.003). In contrast, there were two deaths in the pediatric group, one for each type of bacteremia (CAB and HAB) without a statistical difference in survival rates (91% versus 75%, p = 0.390). The only death in the CAB pediatric group occurred within the first ten days after bacteria isolation. In the univariate analysis, older age (≥ 65-years, p = 0.010), qSOFA score ≥2 (p = 0.025), and CAB (p = 0.004) were associated with increased mortality (Table 3). In the multivariate analysis, a qSOFA score of ≥2 and CAB were predictors of mortality.

**Table 3:** Univariate and multivariate Cox regression analysis of risk factors for mortality in patients with hematological malignancies and *Aeromonas sobria* bacteremia.

Risk factors		Univariate analysis		Multivariate analysis	
		cHR (95% CI)	P-value	aHR (95% CI)	P-value
Age group	Pediatric	reference	-	reference	-
	Adult	3.07 (0.65–14.49)	0.156	2.28 (0.41–12.49)	0.344
Comorbidities	No	reference	-	reference	-
	Yes	1.22 (0.31–4.71)	0.777	0.52 (0.11–2.54)	0.417
qSOFA score	0–1	reference	-	reference	-
	≥2	4.59 (1.22–17.30)	0.025	5.30 (1.12–25.16)	0.036
Type of bacteremia	HAB	reference	-	reference	-
	CAB	9.73 (2.05–46.11)	0.004	8.64 (1.53–48.65)	0.014

aHR, Adjusted Hazar Ratio; cHR, Crude Hazar Ratio; CI, Confidence Interval; qSOFA, Quick Sequential Organ Failure Assessment; HAB, Healthcare-associated bacteremia; CAB, Community-acquired bacteremia.

### Antimicrobial susceptibility testing

Table 4 shows the antimicrobial susceptibility testing results. Overall, *A*. *sobria* was susceptible to most of the antibiotics used; patterns of resistance were found in patients treated with ciprofloxacin in both the adult (4/22, 18%) and pediatric (1/15, 7%) population.

**Table 4:** Susceptible and resistant estimates of antibiotics of patients with hematological malignancies and *Aeromonas sobria* bacteremia in the overall population and according to age group.

Drugs tested	Pediatric patients (n = 15)		Adult patients (n = 22)		All patients (n = 37)	
	Susceptible/tested isolates (%)	Resistant/tested isolates (%)	Susceptible/tested isolates (%)	Resistant/tested isolates (%)	Susceptible/tested isolates (%)	Resistant/tested isolates (%)
Amikacin	14/14 (100)	0/14 (0)	21/22 (95)	1/22 (5)	35/36 (97)	1/36 (3)
Aztreonam	15/15 (100)	0/15 (0)	20/21 (95)	1/21 (5)	35/36 (97)	1/36 (3)
Cefepime	15/15 (100)	0/15 (0)	19/20 (95)	1/20 (5)	34/35 (97)	1/35 (3)
Cefoperazone-sulbactam	-	-	2/2 (100)	0/2 (0)	2/2 (100)	0/2 (0)
Cefotaxime	12/12 (100)	0/12 (0)	14/15 (93)	1/15 (7)	26/27 (96)	1/27 (4)
Ceftazidime	14/14 (100)	0/14 (0)	21/22 (95)	1/22 (5)	35/36 (97)	1/36 (3)
Ceftriaxone	13/13 (100)	0/13 (0)	19/20 (95)	1/20 (5)	32/33 (97)	1/33 (3)
Ciprofloxacin	14/15 (93)	1/15 (7)	18/22 (82)	4/22 (18)	32/37 (86)	5/37 (14)
Imipenem	8/8 (100)	0/8 (0)	9/10 (90)	1/10 (10)	17/18 (94)	1/18 (6)
Levofloxacin	-	-	1/1 (100)	0/1 (0)	1/1 (100)	0/1 (0)
Meropenem	15/15 (100)	0/15 (0)	20/21 (95)	1/21 (5)	35/36 (97)	1/36 (3)
Piperacillin-tazobactam	-	-	1/2 (50)	1/2 (50)	1/2 (50)	1/2 (50)
Trimethoprim-sulfamethoxazole	-	-	1/1 (100)	0/1 (0)	1/1 (100)	0/1 (0)
Vancomycin	1/1 (100)	0/1 (0)	-	-	1/1 (100)	0/1 (0)

## Discussion

Bacteremia caused by the *Aeromonas* species is associated with high mortality rates. However, data regarding the outcomes in patients with malignant hematologic diseases is limited. This study reports the influence on survival of the type of bacteremia (CAB versus HAB) by *A*. *sobria* on patients with malignant hematologic diseases and their clinical course. By collecting data over 17 years, we observed that patients with CAB have an apparent worse survival compared to patients with HAB. Furthermore, we found distinct clinical outcomes on patients with *A*. *sobria* bacteremia based on clinical presentation.

Abdominal pain was the most common presenting symptom of *A*. *sobria* bacteremia in this study, with the gastrointestinal tract as the most common primary source of sepsis. This goes in line with previous reports that describe abdominal pain as the most common presenting symptom [13]. Other common symptoms include diarrhea and fever [8, 9, 11, 14, 19–21], which were also presented in our population.

Our findings add to the literature the 30-day overall survival rates in patients with hematological malignancies and bacteremia with *A*. *sobria*. In contrast to previous reports, we included pediatric and adult patients and analyzed them separately given the difference in the incidence of hematologic malignancies seen according to age group (i.e., acute lymphoblastic leukemia is more common in pediatric than adult patients), and its distinctive clinical course and outcome (i.e., pediatric acute leukemia is often associated to better outcomes compared to acute leukemia during adulthood). Case-fatality rates were higher in adult patients compared to pediatric patients. As opposed to pediatric patients, adults had a higher percentage of pre-existing comorbidities (31.8% versus 6.7%; p = 0.108); this may have influenced the intensity of cancer-directed therapy the adults received, consequently increasing the fatality rates seen in this age group, an observation that has been reported on a previous study [13]. However, in the multivariate analysis, age could not be established as an independent prognostic factor for mortality.

Our study found CAB as a negative prognostic factor for survival in both the univariate and multivariate analysis. This finding differs from previous reports that described either HAB as a risk factor for mortality, or no significant differences in mortality between both types of bacteremia [8, 13]. Community patients presented with higher percentages of septic shock and qSOFA scores in our cohort, implying a higher disease severity. Although we adjusted for qSOFA in the multivariate model, CAB remains a prognostic factor for mortality. We highlight some explanations for this outcome. First, patients with CAB had a higher median age at diagnosis compared to patients with HAB. Second, community patients may have experienced a delay in bacteremia diagnosis. Hospitalized patients are daily assessed through quantification of vital signs and periodic blood work, which increases the likelihood of early detection and subsequent treatment of an evolving infection. In contrast, these procedures are less consistent in the outpatient setting, increasing the disease severity at presentation. Third, patients in the CAB cohort could have had worse performance status or comorbidities with a worse prognosis than patients in the HAB subgroup. These factors might have increased the mortality in the CAB cohort; however, we lack data to corroborate these explanations.

Prior studies have described the clinical course and the associated factors for mortality in patients with bacteremia by *A*. *sobria*. However, we attempted to find the influence of the type of bacteremia in our patient population using conventional methods for survival analysis. One small report drew inference using a parametric statistical test, and others used regression techniques to identify whether certain factors increase the odds of mortality [9, 11]. We opted for a more tailored approach by using non-parametric tests (Fisher’s exact and Mann-Whitney U test) to make inferences on the relatively small count and distribution of our population. Furthermore, we used time-to-event analysis (Kaplan-Meier and Cox regression) to identify the survival differences and prognosis between both types of bacteremia.

Most of the strains in this study were resistant to ciprofloxacin, which correlates with previous findings [8, 13]. It is important to mention that nearly all the tested isolates were susceptible to antibiotics in the pediatric population, most of whom had HAB. Therefore, we can hypothesize that bacteremia caused by resistant strains of *A*. *sobria* happens in patients infected in the community setting, most of whom are adults. All community patients in this study received antimicrobial prophylaxis with levofloxacin upon discharge. Thus, it is unclear to what extent this approach may have contributed to the observed pattern of resistance and survival outcomes.

Our study has some limitations. First, this is a single-center study, hence, our results should be taken with caution. Second, the results of the antimicrobial susceptibility testing in this study were heterogeneous, due to the lack of a standardized protocol regarding which drugs should be tested when *A*. *sobria* is isolated from the bloodstream. However, this report is one of the largest cohorts of patients with *A*. *sobria* infection and hematologic malignancies. *A*. *sobria* bacteremia in these patients is a rare condition that limits the gathering of large sample size and statistical analysis. Third, we were unable to include septic shock in our model because of the violation of the hazard assumption and we could not perform a stratify analysis by septic shock status at presentation given the low count between patients with HAB or CAB. As a result, we used qSOFA as a proxy for severity at presentation. Despite these shortcomings, our chosen Cox regression model did not violate the proportional hazard assumption, making our results reliable. Moreover, we were able to provide survival probabilities and case-fatality rates, in contrast to a prior study that only reported death from any cause [13]. Therefore, our approach gives a more extensive analysis of the risk of mortality and survival prognosis from *A*. *sobria*.

In conclusion, CAB is associated with higher mortality risk and worse OS in individuals with hematologic malignancies and *A*. *sobria* bacteremia. These findings suggest that individuals with CAB present with worse disease severity. Our results should aid clinicians to determine the survival prognosis in this population.
